# HIV controllers, lessons learnt from the lymphoid tissues

**DOI:** 10.1097/COH.0000000000001025

**Published:** 2026-02-28

**Authors:** Andrea Mastrangelo, Riddhima Banga, Matthieu Perreau

**Affiliations:** Divisions of Immunology and Allergy, Lausanne University Hospital, University of Lausanne, Lausanne, Switzerland

**Keywords:** compartmentalization, GALT, HIV persistence, lymph node, T-follicular helper cells, tissue HIV reservoir

## Abstract

**Purpose of review:**

The existence of HIV controllers offers the possibility of identifying specific immune-virological attributes associated with the control of HIV replication, possibly guiding the development of immunological interventions to achieve a functional cure. In the past few years, pioneering studies focused on the study of viral reservoir and immune responses in lymphoid tissues. In the present review, we propose to summarize their findings.

**Recent findings:**

The mechanisms of HIV control in tissues may present peculiar features. Despite efficient viral suppression *in vitro*, HIV-specific CD8 T cells from lymphoid tissues express lower levels of the classical cytotoxic molecules (Granzyme B and Perforin) directly *ex vivo*, suggesting that alternative mechanism(s) may operate in these compartments. Interestingly, tissue-resident CD8 T cells from multiple tissues express an array of alternative granzymes, whose contribution to the control of HIV replication remains to be established. NK cells also display peculiar features in lymphoid tissues, including lower levels of classical cytotoxic molecules and increased expression of CXCR5, highlighting the complexity of tissue-specific immunity in the context of HIV.

**Summary:**

The immunological mechanisms associated with the control of HIV replication in tissues remain to be fully identified and may involve multiple mediators acting through distinct mechanisms. Recent evidence suggests that the control of HIV replication in lymphoid tissues probably depends on an appropriate positioning of effector cells in the tissue microenvironment, and may be mediated by different mechanisms depending on the levels of viral production and the body compartment.

## INTRODUCTION

Achieving an HIV cure needs to overcome numerous obstacles associated with both the peculiar virology of HIV and the impaired host antiviral immune response observed in more than 99% of persons with HIV (PWH) in absence of appropriate antiretroviral therapy (ART). Indeed, one of the major barriers to HIV eradication resides in the capacity of the virus to establish a reservoir very rapidly. The HIV reservoir, defined by the presence of cells containing inducible replication competent virus that can persist for years, is highly complex in terms of cellular composition, that is, CD4 T cells, macrophages, and dendritic cells [1–4], in terms of transcriptional activity [5,6] and in terms of tissue distribution, as HIV-infected cells can be found in multiple compartments and tissues (e.g. blood [1], lymph nodes [7,8]), intestinal tract [9], central nervous system [10]), complicating eradication efforts. Moreover, the HIV reservoir is also heavily dynamic in all these dimensions [11]. In addition, HIV replication remains uncontrolled in most PWH, leading to persistent antigen exposure and progressive functional impairment of the immune system [12,13], which is not fully restored following ART introduction [13]. However, early in the epidemic, interesting reports revealed that some PWH did not progress to AIDS [14]. These individuals were referred to as long-term nonprogressors (LTNP), and were characterized by preserved CD4 T-cell counts (exceeding 500 cells/μl of blood) [15]. As viral load testing methods became more sensitive and more specific in the mid-2000s, researchers began distinguishing LTNP based on HIV viremia, giving rise to the more recent categories of HIV controllers, i.e. viremic controllers [16], able to achieve a partial control of HIV replication and defined by a viral load between 50 and 2000 HIV RNA copies/ml and elite controllers [16], with undetectable viral load (<50 HIV RNA copies/ml). Interestingly, some HIV controllers may progressively lose the control of HIV replication over time (transient controllers) [17–19], while others may control HIV replication for an extensive period of time (exceptional controllers) [20]. In addition to the above mentioned ‘natural controllers’, additional groups of individuals recently emerged from studies of structured treatment interruption with or without immunological interventions [21], and were, therefore, referred to as posttreatment controllers (PTCs) [22] or postintervention controllers (PICs) [23].

Although the control of HIV replication is a rare phenomenon, it offers the possibility of identifying specific immunovirological signatures representing a fundamental step to guide the development of future immunological interventions aiming to prevent HIV infection or achieve a functional cure. 

**Box 1:**
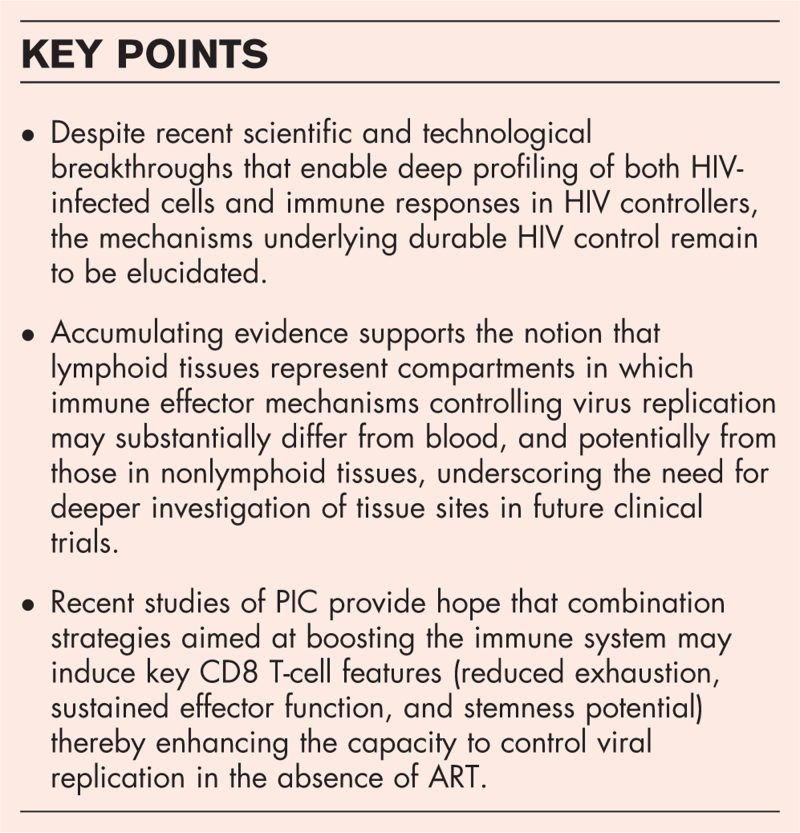
no caption available

## VIROLOGICAL CHARACTERIZATION OF HIV CONTROLLERS IN LYMPHOID TISSUES

Despite initial reports suggesting that HIV controllers might have been infected with ‘attenuated’ viral variants, intrinsically displaying reduced viral fitness [24], accumulating evidence demonstrated that a vast majority of viral variants observed in HIV controllers were undistinguishable from those observed in HIV progressors [25], opening the way to the investigations of the virological features associated with the natural control of HIV replication. The field, based on sample accessibility, naturally oriented its investigations on the study of HIV reservoir of HIV controllers in blood, which was thought to provide an appropriate window into the reservoir cells and immune activity at the predominant sites of viral replication, that is, the lymphoid tissues. These studies highlighted that the HIV reservoir of HIV controllers as measured in blood was significantly smaller in size, in terms of both frequencies of cells containing total integrated HIV DNA [26] and frequencies of cells containing intact proviruses [27], as compared to untreated viremic chronic progressors or ART-treated aviremic PWH. However, study of peripheral blood only provides a limited view on the reservoir features and dynamics in PWH, as most of the infected cells in either noncontrollers or HIV controllers are located in lymphoid tissues. Indeed, pioneering studies indicated that HIV replication and persistent viral transcription occurs predominantly in secondary lymphoid tissues, that is, spleen, lymph nodes, and gut-associated lymphoid tissues (GALT) [9,28] in absence of ART. In addition, lymphocyte populations located in lymphoid tissues differ from their blood counterparts not only quantitatively, but also in their spatial organization in different zones of secondary lymphoid tissues [i.e. the paracortex, the interfollicular region (T/B border) or the B-cell follicles], which dictates functional and anatomical differentiation of CD4 T cells populations and may impact HIV replication dynamic. In this context, we and others showed that lymph node PD-1^+^ CD4 T cells, including T-follicular helper (Tfh) cells, served as the major CD4 T-cell compartment for viral production and transcription, although HIV transcription could also be detected in nonfollicular CD4 T cells [7,29]. Two years later the group of Louis Picker showed that productive SIV infection of elite controller rhesus macaques was restricted to cells located in follicular areas [30] during efficient viral control. However, depletion of CD8^+^ cells resulted in a transient increase in productively SIV-infected Tfh cells and to the re-distribution of productive SIV infection to CD4 T cells located in the extra-follicular areas [30]. This study largely supported the concept of an ongoing ‘battle’ between the host immune response and SIV/HIV-infected cells in lymphoid compartments, suggesting that during spontaneous HIV/SIV control the immune pressure can reduce viral replication in lymphoid tissues, operating with different efficacy in the extra-follicular and follicular areas.

Interestingly, recent studies showed that intact proviral HIV genomes detected in elite controllers were predominantly located in DNA regions with poor transcriptional activity, particularly in centromeric satellite DNA or zinc-finger genes [27,31]. This observation suggested that cells with intact provirus may harbor limited capacity to be induced in HIV controllers and the unique HIV reservoir landscape identified in HIV controllers may result from a selection process, most likely mediated by the immune system that eliminates HIV-infected cells [31]. Notably, longitudinal assessment of paired blood and lymph node samples from elite controllers also observed a reduction of intact proviruses in both compartments, and lower frequencies of intact proviruses within lymph node tissue relative to blood [32^▪^]. Interestingly, a recent study revealed that this process of reservoir ‘trimming’ may not occur at the same extent in all elite controllers, and that its efficacy may be crucial in dictating long-term outcomes of immune-mediated viral control [33]. Indeed, the intact HIV proviral DNA was more frequently detected in permissive genic euchromatic positions in peripheral blood mononuclear cells (PBMCs) collected from HIV controllers before that these individuals spontaneously lost the control of HIV replication, highlighting the possibility that a change in the HIV reservoir landscape may precede the loss of viral control in HIV controllers [33].

Taken together, these studies revealed the peculiar HIV reservoir landscape of HIV controllers and the need to investigate the determinants of HIV control not only in blood but also within organized and nonorganized lymphoid tissues, where unique immune populations and antiviral responses may reside.

## CELLULAR IMMUNE RESPONSES IN TISSUES

Numerous genetic studies demonstrated an association between the expression of specific class I Human Leukocyte Antigen (HLA) alleles (i.e. B*57 or B*27, referred to as ‘protective’ alleles) and the control HIV replication [34–37], while associating other HLA class I molecules (i.e. B*08 or B*35) with a faster HIV disease progression (reviewed in reference [35]). These observations strongly supported a preponderant role played by the cellular immunity (CD8 T cell and/or NK cells) as a major immunological determinant of natural control of HIV replication. Nonetheless, the expression of ‘protective’ HLA alleles is neither necessary nor sufficient to control HIV replication, as untreated viremic chronic progressors can express ‘protective’ HLA alleles [34,35], and on the other side, up to one-third of HIV controllers do not express the aforementioned ‘protective’ HLA alleles [35,38]. These observations suggest that the capacity of cellular immunity to suppress viral replication may not be entirely genetically predetermined and may also be influenced by other factors, potentially amenable to specific interventions [39^▪▪^,^40^^▪▪^]. Additionally, these findings also underscore the potential role of additional responses, such as innate and humoral immune responses, in viral suppression (reviewed in references [41,42]).

The crucial role played by the cellular arm of the immune system in the control of SIV/SHIV-replication was further demonstrated by cellular depletion studies, where the depletion of both CD8 T cells and NK cells [30] or the selective depletion of CD8 T cells [43,44] in SIV or SHIV-infected nonhuman primates naturally controlling SIV/SHIV replication was consistently associated with viral rebound. To identify specific features associated with the control of HIV replication, numerous studies compared the phenotype and functions of HIV-specific CD8 T cells of HIV controllers versus the one of progressors. These studies showed that HIV-specific CD8 T cells from HIV controllers appeared to be less exhausted (lower level of expression of immune checkpoint molecules such as PD-1 [12,13] and TIGIT [45]), produced multiple cytokines such as IFN-γ, IL-2, and/or TNF [46,47], proliferated upon antigen stimulation [48] and expressed cytotoxic granules containing perforin and granzyme B [49,50], defining the concept of ‘polyfunctionality’ [47,51].

More recently, the expression of specific transcription factors, such as TCF1 [52], has been associated with viral control, and recent studies observed that PIC maintaining viral suppression after anti-HIV broadly-neutralizing antibody (bNAbs) administration displayed enhanced CD8 T-cell responses marked by stem-like features, TCF1 expression, and robust expansion against rebound virus [39^▪▪^,^40^^▪▪^]. Notably, whether these enhanced functional attributes were the causes, or the consequences of the virological control observed in HIV controllers remains an open question.

As HIV/SIV preferentially replicates in lymphoid compartments, the phenotypic and functional profiles of HIV/SIV-specific CD8 T cells were also studied in lymphoid tissues. Multiple lines of evidence underscored that HIV-specific CD8 T-cell responses at a tissue level may show peculiar phenotypic and functional features, distinct from and not necessarily detectable in peripheral blood, and that CD8 T cells’ localization and spatial positioning within tissues of HIV controllers play a critical role to adequately control HIV replication [49,53–55]. Notably, in GALT, high frequency of HIV-specific CD8 T cells of HIV controllers exhibiting polyfunctional (defined by coordinate expression of IFN-γ, IL-2, and TNF-α, MIP1b and CD107a) [56,57] profiles were proposed to strategically position within the lamina propria and other mucosal sites as compared with those of chronic progressors [56,57]. In lymph nodes, multiple reports observed that CXCR5-expressing HIV-specific CD8 T cells with the capacity to locate in follicular areas can be detected [54,58] and may be potentially involved in viral suppression in HIV controllers [49,54]. Moreover, the frequency of lymph node-resident (CD69^+^) HIV-specific CD8 T cells, able to provide rapid local response in lymph nodes, was also found to be increased in HIV controllers, pointing at tissue immune responses as crucial determinants of viral suppression [59].

In addition to the investigation of the spatial distribution of CD8 T cells, several studies were conducted in lymph nodes collected from either PWH or NHP models to better appreciate the potential mechanism of viral suppression occurring in lymphoid tissues of controllers. Nguyen and colleagues performed a transcriptomic analysis of lymph node HIV-specific CD8 T cells and observed a distinct transcriptional signature in elite controllers, characterized by reduced expression of inhibitory receptors and cytolytic molecules (Perforin^+^/Granzyme B^+^) and increased expression of cytokines and ribosomal protein subunits [53]. These characteristics were probably associated with an efficient protein translational activity in HIV controllers, and suggested a preferentially noncytolytic mechanism of control [53]. Notably, previous evidence from non-human primates (NHPs) also suggested that HIV replication may be suppressed by CD8 T cells using a noncytolytic mechanism [44,60]. Interestingly, Collins *et al.* proposed that HIV-specific CD8 T cells could control ongoing viral replication in lymph nodes through conventional cytotoxic mechanism (perforin and Granzyme B) despite low ex-vivo expression, suggesting that, in lymphoid tissues, this machinery may operate only following a first cellular proliferation step [49]. In this view, HIV-specific CD8 T cells may acquire maximal cytotoxic capacity after few days post antigen encounter, and exert effector functions after migrating in close proximity of HIV-infected cells located in the follicles [49,61]. Alternatively, recent evidence suggests that tissue-resident and lymph node-resident CD8 T cells may display substantially lower levels of perforin and Granzyme B *ex vivo* but may be enriched in alternative granzymes not particularly expressed in cells located in peripheral blood [62^▪▪^]. Whether these atypical granzymes may be involved in HIV suppression in tissues remains, however, to be investigated. In this view, recent evidence suggested that GZMK-expressing cells may be inversely correlated to viral load in the SIV model [63^▪▪^].

On the basis of what precedes, current evidence suggests that several viral suppression mechanisms may contribute to control viral replication in lymphoid tissues. HIV-specific CD8 T cells of HIV controllers may harbor yet unidentified nonclassical cytotoxic effector functions and/or may suppress HIV replication using noncytotoxic function(s). Alternatively, lymph node HIV-specific CD8 T cells may lack immediate effector mechanisms, but retain the capacity to proliferate and acquire Perforin^+^/Granzyme B^+^ cytotoxic granules to mediate viral control. Of note, these mechanisms may not be mutually exclusive, and their relative contribution to viral suppression may vary depending on several factors, including levels of viral replication, the stage of the HIV infection (acute vs. chronic) and/or the body compartment/tissue.

Based on the NHP depletion studies, the contribution of NK to viral control was also investigated in HIV controllers. These studies revealed that HIV controllers harbored increased frequencies of blood NK cells (CD56^+^/CD16^-^) composed with distinct functional subsets [64]. Interestingly, in PTCs, the frequencies of NKp46^+^/NKp30^+^ NK cells were associated with lower levels active viral reservoir [65], suggesting that this cellular population may contribute to viral control. Furthermore, PTCs had higher levels of activated NK cells before analytical treatment interruption, the frequencies of which correlated with lower levels of rebound viremia, suggesting a role for NK cells in suppressing HIV replication after ART discontinuation [22]. Limited number of studies investigated the antiviral role of NK cells in lymphoid tissues. Notably, in NHP models, lymph node CXCR5^+^ NKs harboring a specific transcriptomic profile have been linked to viral control and a lack of disease progression in SIV-infected African green monkeys [66]. In humans, however, the data on lymph node NK cell spatial localization and effector function during HIV infection remain rather limited. One study observed that a CXCR3^+^ NK cell subset (T-bet^+^ IFN-γ^+^), localized in interfollicular regions, may promote Th1 polarization and antiviral activity within these areas [67]. In contrast, a ‘follicular’ CXCR5^+^ NK cell subset, characterized by elevated IFN-γ, CD69, CD107a, and β-chemokines but relatively weak cytotoxicity compared to extra-follicular NK cells, was shown to be enriched in HIV infection and inversely associated with HIV DNA and RNA levels in lymph nodes [68]. Interestingly, recent data suggested that poorly cytolytic CD56^bright^ NK cells, continuously recirculating between blood and lymph nodes, represent the preferential subtype of NK cells found in lymphatic tissues. CD56^dim^ NK cells, harboring an increased cytolytic potential, are preferentially retained in peripheral blood, echoing previous observations on CD8 T cells [69], and may access lymphoid tissues in case of inflammation [70^▪▪^]. The relative contribution of either subset to viral suppression in the context of HIV remains to be fully elucidated.

Collectively, these observations underscore the need for a deeper characterization of NK-cell phenotypes, transcriptional states, and tissue and compartment-specific functions to better understand their potential role in controlling HIV replication.

## CONCLUSION

The control of HIV replication is likely mediated by multiple components of the immune system. As the precise mechanism(s) remain(s) largely undefined, studies in spontaneous controllers that may capture the earliest interactions between the emerging virus and the immune system will be critical. In addition, characterizing the timing, quality, and tissue-specific distribution of these responses during early HIV/SIV infection and longitudinally across multiple compartments, including lymphoid and other anatomical sites, will be essential to understand how natural control is established and maintained. Furthermore, recent studies of PICs, who controlled viral replication following bNAb interventions, offer the additional hope that beyond genetic factors that may contribute to control in some individuals, timely immunological support to the immune system through interventions may limit CD8 T-cell exhaustion and preserve their functionality, including effector functions and stem-like potential [40^▪▪^]. This preserved functionality could enable tissue and compartment-specific effector responses, locally supported by resident immune cells to control HIV replication in tissues without ART.

## Acknowledgements

*None*.

### Financial support and sponsorship

*This work was supported by Freedom Forever research grant to M.P. and A.M.*

### Conflicts of interest

*There are no conflicts of interest.*

## REFERENCES AND RECOMMENDED READING

Papers of particular interest, published within the annual period of review, have been highlighted as:

▪ of special interest
▪▪ of outstanding interest
